# Which score should be used for posttraumatic multiple organ failure? - Comparison of the MODS, Denver- and SOFA- Scores

**DOI:** 10.1186/s13049-016-0321-5

**Published:** 2016-11-03

**Authors:** Matthias Fröhlich, Arasch Wafaisade, Anastasios Mansuri, Paola Koenen, Christian Probst, Marc Maegele, Bertil Bouillon, Samir G. Sakka

**Affiliations:** 1 Department of Trauma and Orthopedic Surgery, University of Witten/Herdecke, Cologne-Merheim Medical Center (CMMC), Ostmerheimerstr. 200, D-51109 Cologne, Germany; 2 Institute for Research in Operative Medicine (IFOM), University of Witten/Herdecke, Ostmerheimerstr. 200, D-51109 Cologne, Germany; 3 Department of Anaesthesiology and Operative Intensive Care Medicine, Medical Centre Cologne-Merheim, University of Witten/Herdecke, Cologne, Germany

**Keywords:** Trauma, Multiple organ failure, Multiple organ dysfunction, Sofa score, Denver Score

## Abstract

**Background:**

Multiple organ dysfunction and multiple organ failure (MOF) is still a major complication and challenge in the treatment of severely injured patients. The incidence varies decisively in current studies, which complicates the comparability regarding risk factors, treatment recommendations and patients’ outcome. Therefore, we analysed how the currently used scoring systems, the MODS, Denver- and SOFA Score, influence the definition and compared the scores’ predictive ability.

**Methods:**

Out of datasets of severely injured patients (ISS ≥ 16, Age ≥ 16) staying more tha 48 h on the ICU, the scores were calculated, respectively. The scores’ predictive ability on day three after trauma for resource requiring measurements and patient specific outcomes were compared using receiver-operating characteristics.

**Results:**

One hundred seventy-six patients with a mean ISS 28 ± 13 could be included. MODS and SOFA score defined the incidence of MOF consistently (46.5 % vs. 52.3 %), while the Denver score defined MOF in 22.2 %. The MODS outperformed Denver- and SOFA score in predicting mortality (area under the curve/AUC: 0.83 vs. 0.67 vs. 0.72), but was inferior predicting the length of stay (AUC 0.71 vs.0.80 vs.0.82) and a prolonged time on mechanical ventilation (AUC 0.75 vs. 0.81 vs. 0.84). MODS and SOFA score were comparably sensitive and the Denver score more specific in all analyses.

**Conclusions:**

All three scores have a comparable ability to predict the outcome in trauma patients including patients with severe traumatic brain injury (TBI). Either score could be favored depending weather a higher sensitivity or specificity is targeted. The SOFA score showed the most balanced relation of sensitivity and specificity. The incidence of posttraumatic MOF relies decisively on the score applied. Therefore harmonizing the competing scores and definitions is desirable.

## Background

Despite all improvements in trauma care during the last decades, post-injury multiple organ failure (MOF) remains a major complication and challenge in severely injured patients [1]. During the post-traumatic hospital course, it has been described as “resource- intensive, morbid and lethal” and is considered as the main cause of late post-injury mortality [2, 3]. Furthermore, MOF causes up to 30 % among the possibly preventable deaths [4].

According to our groups’ previous work, the incidence of posttraumatic MOF in severely injured patients increased during the last decade accompanied by a decreasing case fatality rate [5]. However, the incidences of MOF in different comparable studies varied decisively and ranged from 6 to 42 % [1, 2, 5–7]. On the one hand, differences in inclusion criteria, patient’s treatment and trauma systems may explain some of these differences. However, all of these studies originated from developed trauma systems and focused on severely injured patients. On the other hand, in most recent publications, three different scores defining MOF were used: the Sequential Organ Assessment Score (SOFA), the Marshall Multiple Organ Dysfunction Score (MODS), and the Denver score. Although these three scores define the same syndrome, there are substantial differences in selection and assessment of observed organ systems. Presumably, the selected score might have a significant influence on the observed MOF incidence and complicates comparing observed incidence rates.

Originally these scores were not developed to predict patients’ outcome. Since MOF is a major complication during the post-traumatic treatment, the scores’ predictive value on patients’ outcome is clinically relevant during daily trauma care. Furthermore, the understanding of scoring MOF is valuable in research, for example, in stratifying and including patients for clinical trials that include the endpoint MOF. Up to date, there has not been a comparison of the Denver, MODS and SOFA scores applied on the same data set. Therefore, in the present study we aimed to compare these three most frequently used MOF scores for their ability to predict the outcome in severely injured patients.

## Methods

### Study population

**Fig. 1:**
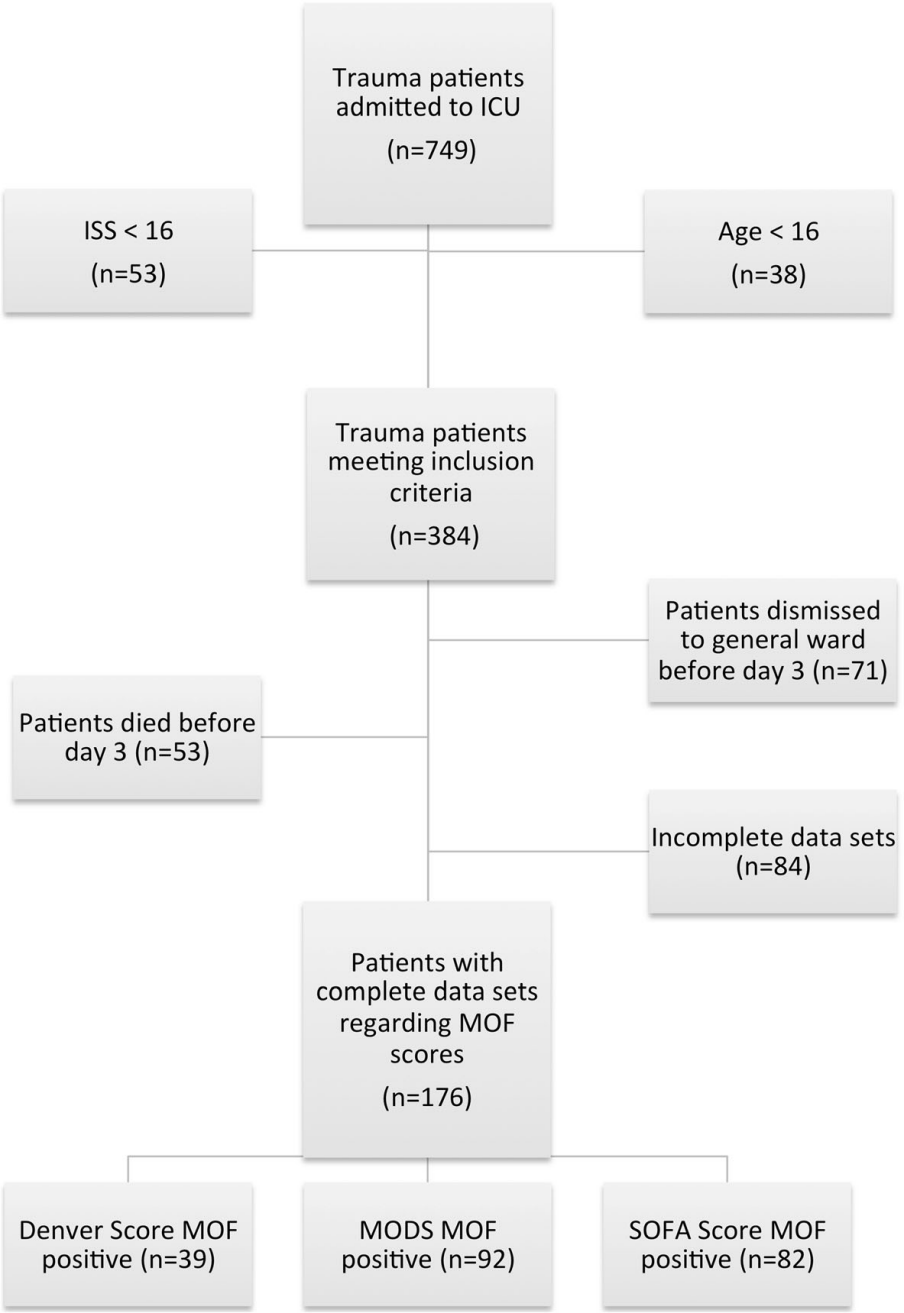
Study patient numbers

All severely injured patients (*n* = 749), who were admitted to the intensive care unit (ICU) of our Level I Trauma Center between 2011 and 2013, were eligible for further analysis. Inclusion criteria were a relevant trauma load displayed by an ISS (Injury Severity Score) ≥ 16, age ≥ 16 years and a length of stay on ICU for more than 48 h. Patients with incomplete data sets regarding one of the scores were excluded. Detailed patient numbers are displayed in Fig. 1.

Patient characteristics such as demographics and comorbidities were recorded at hospital admission. Vital parameters and laboratory data were recorded daily through the ICU stay. Injury pattern including injury mechanism and severity displayed by ISS and New Injury Severity Score (NISS) were assessed. Using the Revised Injury Severity Classification (RISC II) the predicted mortality was calculated [8]. Clinical events were recorded until death or hospital discharge. The local ethics committee of the Cologne Merheim Medical Center approved the study. Patient records and information were anonymised prior to analysis. According to the ethics committee individual patient consent was not required.

### Multiple organ failure scores

Multiple organ failure was defined according to three currently used scores, the SOFA-, MODS and Denver score (Table 1).

**Table 1:** Summery of the Denver Score, SOFA Score and MOD Score

Dysfunction	Grade 0	Grade 1	Grade 2	Grade 3	Grade 4
Denver Score					
Pulmonary, PaO_2_/FIO_2_, [mmHg]	>208	208–165	165–83	<83	
Hepatic, bilirubin, [μmol/L]	<34	34–68	69–137	>137	
Renal creatinine, [μmol/L]	<159	160–210	211–420	>420	
Cardiac inotropes^a^	No inotropes	Only 1 inotrope at a small dose	Any inotrope at moderate dose or >1 agent at small dose	Any inotrope at large dose or >2 agents at moderate dose	
SOFA score					
Pulmonary, PaO_2_/FIO_2_, [mmHg]	>400	≤400	≤300	≤200	≤100
Coagulation, platelet count, [×10^3^/μL]	>150	≤150	≤100	≤50	≤20
Hepatic, bilirubin, [μmol/L]	≤20	20–32	33–101	102–204	>204
Cardiovascular, inotropes^b^ in μg/kg/min	No hypotension	Mean arterial pressure <70 mmHg	Dopa ≤5 or any Dobu dose	Dopa >5 or Epi ≤0.1 or Nor ≤ 0.1	Dopa >15 or Epi >0.1 or Nor >0.1
Renal, creatinine, [μmol/L]	<110	110–170	171–299	300–440	>440
Central nervous system, GCS	15	13–14	10–12	6–9	<6
MODS					
Pulmonary, PaO_2_/FIO_2_, [mmHg]	>300	226–300	151–225	76–150	≤51
Renal, creatinine, [μmol/L]	≤100	101–200	201–350	351–500	>500
Hepatic, bilirubin, [μmol/L]	≤20	21–60	61–120	121–240	>240
Cardiovascular, PAR^c^	≤10.0	10.1–15.0	15.1–20.0	20.1–30.0	>30.0
Coagulation, platelet count, ×10^3^/μL	>120	81–120	51–80	21–50	≤20
Central nervous system, GCS	15	13–14	10–12	6–9	<6

^a^Inotrope doses (in μg/kg/min): vasopressin: small <0.03, moderate 0.03–0.07, large >0.07; dopamine: small <6, moderate 6–10, large >10; dobutamine: small <6, moderate 6–10, large >10; epinephrine: small <0.06, moderate 0.06–0.15, large >0.15; norepinephrine: small <0.11, moderate 0.11–0.5, large >0.5

^b^ *Dopa* Dopamine, *Dobu* Dobutamine, *Epi* Epinephrine, *Nor* Norepinephrine

^c^PAR = Heart Rate × Central Venous Pressure/Mean Arterial Blood Pressure

The SOFA score, initially used to assess critically ill ICU patients and secondly validated for trauma patients, is composed of scores from six organ systems, graded from 0 to 4 according to the degree of dysfunction or failure [9–11]. A score of 3 or greater for one of the organ systems was defined as a failure of this organ. In both the initial description and the evaluation of the SOFA score by Vincent et al. [9, 10] there is no statement on when to define multiple organ failure. As frequently used previously, in the current study MOF was defined as organ failure (score ≥ 3 points) of at least two of the listed organs or systems [5, 12].

The Marshall Multiple Organ Dysfunction Score (MODS) assesses the same six organ systems using slightly different values for four grades of organ dysfunction. Most obvious is the difference in grading the cardiovascular system. In contrast to the surrogate parameter, use of inotropic medication, a composite measure, the pressure-adjusted heart rate (PAR) is used. The PAR is calculated by heart rate (HR) multiplied by the ratio of the central venous pressure (CVP) to the mean arterial pressure (MAP) [13]. The total score, ranging from 0 to 24, arises from the sum of all single organ scores using the first measured value of the day. Marshall et al. did not define a specific cut-off for the diagnosis of MOF. Instead, the authors associated score ranges with mortality rates [13]. However, previous studies, that validated the MODS, have defined a score of more than 5 either for one day or two consecutive days to define the presence of MOF [1, 14].

The Denver score has been specifically developed to assess posttraumatic organ failure excluding severe traumatic brain injury (TBI). The score rates four organ systems on a scale from 0 to 3 (Table 1). In difference to the previously presented scores, the Denver score does not include a grading of the hematologic system and the CNS. The Denver score defines MOF as a score of more than 3 occurring more than 48 h after injury [14, 15].

For calculation of all scores, daily laboratorial and physiological values were used. Due to the comparability of the results, for all scores the worst daily values were used. Daily through the ICU stay, multiple organ failure status was defined as recommended by the authors. However, we revisited the previously described cut-off points using receiver operating characteristic (ROC) curves. Reversible physiologic derangements during the early posttraumatic treatment influence the scores’ grading, but do not represent a substantial organ failure [16]. However, a prediction of the outcome as soon as possible after trauma would be desirable. Therefore and in accordance with previous validations, we used MOF score values on day three after trauma for further analysis and prediction of outcome [14, 17].

### Patient adverse outcomes

The scores were compared by evaluating the scores’ association with patient adverse outcomes, which were ICU length of stay (LOS), days with mechanical ventilation (MVD), ventilator free days (VFD) and hospital mortality. VFDs were calculated as days without mechanical ventilation within 28 days after the injury to account for patients that died early and accordingly had less MVDs [18]. As LOS, MVDs and VFDs were not normally distributed, these outcome parameters were dichotomized for further analysis: 1. ICU LOS and mechanical ventilation up to seven days or longer; 2. ventilator free days of more or less than 21 days. The cut-off points of seven days for LOS and MVD and 21 days for VFD, respectively, were chosen to depict a complicated course during the ICU stay. Furthermore, this stratification allows a comparison with previous validations of either two of the scores, respectively [14, 17]. Sepsis was defined according to the criteria of Bone et al. [19].

### Statistical analysis

Data are presented as mean ± standard deviation (SD) (range of values) for continuous variables or percentages for categorical variables. For the comparison of the performance of the SOFA-, MODS and Denver-Score in predicting patient’s adverse outcomes, the area under the receiving operating characteristics curve (AUROC) was calculated with LOS, MVD, VFD and hospital mortality as the state variables. The comparison of two areas under the receiving operating characteristics curve was based upon the 95 % confidence interval for each curve. For all statistical analyses, a probability of less than 0.05 was considered to be statistically significant. All data were analysed by using IBM SPSS 22 (IBM Corporation, IBM Inc., Armonk, NY, USA).

## Results

In an observation period of three years, 176 severely injured trauma patients remained eligible for further analysis with complete data sets to calculate the Denver-, MODS and SOFA - Score. In the final cohort, patients had a mean age of 53 ± 21 (range: 16–91) years, were predominantly male (67 %) and sustained mainly blunt trauma (96.9 %). Patients were severely injured with a mean ISS of 28 ± 13 (range: 16–50). Severe TBI (AIShead ≥ 3) and thoracic trauma (AISthorax ≥ 3) were observed in 119 and 89 patients, respectively, while severe abdominal and skeletal injuries were less frequent. Out of the final cohort, 32 patients (18.2 %) died after mean 10.2 ± 11.7 (range: 4–29) days after injury. Detailed patient demographics and injury scoring are presented in Table 2. Within the final cohort, there were 32 deaths (18.2 %). Cause of mortality included failure of several organs (28 %), respiratory failure (22 %), failure of cerebral functions (22 %), and sepsis (12 %). In 16 % of the cases, cause of mortality was not documented. Outcome parameters are presented in Table 3.

**Table 2:** Basic demographic data and injury severity and pattern of all patients and patients defined as having MOF by the respective score. Abbreviations: AIS, abbreviated injury scale; ISS, injury severity score; RISC, Revised Injury Severity Classification Score; SD, standard deviation

	All Patients	Denver Score Positive for MOF	MODS Positive for MOF	SOFA Score Positive for MOF
Demographics				
n (total, % of all)	176 (100)	39 (22.2)	92 (52.3)	82 (46.5)
Male (n, %)	111 (63.1)	30 (76.9)	64 (69.6)	57 (69.5)
Age (years; mean ± SD)	53.1 ± 20.9	54.5 ± 19.3	52.2 ± 20.8	52.6 ± 21.3
Blunt trauma (n, %)	170 (96.6)	37 (94.9)	90 (97.8)	79 (96.3)
ASA (mean ± SD)	1.7 ± 0.9	1.9 + 1.1	1.7 ± 0.9	1.8 ± 0.9
Injury Severity				
ISS (points; mean ± SD)	27.6 ± 12.6	34.6 ± 17.5	31.2 ± 14.5	32.5 ± 15.3
NISS (points; mean ± SD)	35.4 ± 14.2	41.4 ± 17.0	40.3 ± 14.6	41.2 ± 16.1
RISC (points; mean ± SD)	16.4 ± 24.7	21.9 ± 30.5	20.6 ± 27.5	22.1 ± 30.1
AIS Head > = 3 points (n; %)	119 (67.6)	29 (74.4)	74 (80.4)	65 (79.3)
AIS Thorax > = 3 points (n; %)	89 (50.6)	22 (56.4)	46 (50.0)	41 (50.0)
AIS Abdomen > = 3 points (n; %)	23 (13.1)	3 (7.69)	10 (10.7)	9 (11.0)
AIS Pelvis/Extremities > = 3 points (n; %)	36 (20.5)	9 (23.1)	20 (21.7)	15 (18.3)

**Table 3:** Outcome of all patients and patients defined as having MOF by the respective score

	All Patients	Denver Score Positive for MOF	MODS Positive for MOF	SOFA Score Positive for MOF
Outcome				
Mortality (n; %)	32 (18.2)	14 (35.9)	26 (28.3)	29 (35.4)
ICU LOS (days; mean ± SD)	14.1 ± 12.6	21.3 ± 14.8	20.7 ± 13.1	19.5 ± 14.1
Ventilator days (days; mean ± SD)	8.5 ± 10.3	14.5 ± 11.6	14.4 ± 10.5	13.7 ± 11.1
Ventilator free days (days; mean ± SD)	16.4 ± 11.3	8.4 ± 9.4	9.5 ± 9.0	8.4 ± 8.9
Sepsis (n; %)	62 (35.2)	22 (56.4)	52 (56.5)	43 (52.4)

*ICU* intensive care unit, *LOS* length of stay

Depending on the score used, the number of patients defined as having MOF differed decisively. While MODS and SOFA - Score accounted for a comparable number of patients (92, 52.3 %; 82, 46.5 %), the Denver- Score defined only 39 patients (22.2 %) as having MOF. Neither demographic data nor injury severity differed significantly between the respective score - groups. Regardless of the score applied, MOF patients were more severely injured displayed by an increased ISS, and a higher ratio had severe head injuries compared to the whole cohort (Table 2).

As expected, patients having MOF had a poor outcome. Regardless of the score applied, MOF patients required a longer ICU LOS and more days on mechanical ventilation, while the length of the inpatient treatment did not differ compared to all patients. As could be expected, mortality was higher when patients were labelled as having MOF regardless of the score applied. However there was no difference in mortality between the MOF groups.

**Fig. 2:**
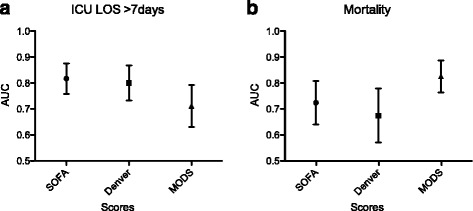
Graphic Representation of the performance analysis of MODS, Denver and SOFA score regarding (**a**) intensive care unit length of stay of more than 7 days and (**b**) hospital mortality displayed as Area Under the Receiver Operating Curve ± 95 % confidence interval

The analysis of the sensitivity and specificity regarding (a) patient specific adverse outcomes such as mortality and ventilator free days and (b) resource - requiring measurements such as ICU LOS and days on mechanical ventilation revealed some differences between the scores (Table 4). In predicting VFD, MODS and SOFA score showed a better relation of sensitivity and specificity compared with the Denver score without differences in the AUC. The MODS convinced with the best sensitivity and highest AUC in predicting mortality, while the Denver Score showed poor sensitivity but good specificity (Fig. 2).

**Table 4:** Performance analysis of MODS, Denver and SOFA score regarding resource requiring and patient specific outcomes. AUC, Area under the receiving operating characteristics curve; CI, confidence interval

	Sensitivity	Specificity	AUC	95 % CI
ICU Length of Stay >7d				
SOFA Score	0.77	0.83	0.82	0.75–0.89
Denver Score	0.53	0.84	0.80	0.73–0.87
MODS	0.61	0.83	0.71	0.63–0.79
Mechanical Ventilation >7 days				
SOFA Score	0.87	0.74	0.84	0.78–0.89
Denver Score	0.34	0.87	0.81	0.75–0.88
MODS	0.70	0.71	0.75	0.67–0.82
Mortality				
SOFA Score	0.81	0.54	0.72	0.64–0.81
Denver Score	0.44	0.83	0.67	0.57–0.78
MODS	0.91	0.63	0.83	0.76–0.89
Ventilator free days > 21 days				
SOFA Score	0.83	0.83	0.89	0.84–0.94
Denver Score	0.59	0.88	0.84	0.78–0.90
MODS	0.76	0.87	0.88	0.82–0.93

Predicting prolonged ICU LOS and days on mechanical ventilation, the SOFA score surpassed substantially the Denver score, but regarding the overall performance, both scores outperformed the MODS in the AUC (Fig. 2).

## Discussion

Originally, the MODS, Denver- and SOFA Score were created for defining the presence of MOF. The three scores differ obviously in their components as the MODS and SOFA score consider the CNS and coagulation system. This might contribute to the increased incidence of MOF, especially in the presence of TBI. As TBI is associated with an increased mortality in trauma patients, this could furthermore influence the scores’ predictive value. Comparing the three scores, the components weighting has to be recognised since the Denver- and SOFA score grade the cardiovascular system using a surrogate parameter (use of inotropic medication) while the MODS depicts physiologic parameters. Despite the interest in an accurate definition of this syndrome, the scores’ ability to predict patients’ adverse outcomes and resource utilization after severe trauma is of clinical relevance. The presented study revealed substantial differences between the scores in sensitivity and specificity, which lead to pronounced variations in the assessed incidence rates of MOF and consecutively in the scores’ predictive values.

The observed incidence of 22 to 52 % according to the applied score appears comparable to previous studies. In a large registry analysis, the MOF incidence was 32.7 % using to the SOFA score [5]. Using one data set, Sauaia et al. described an incidence of 49.7 % for the MODS and 22.2 % for the Denver score [14]. However, all of these numbers appear high compared to clinical experience. In a study comparing the presence of MOF defined by experienced intensive care physicians to the performance of different scores, the clinically defined incidence rate was 26 % and was significantly lower than defined by the scores [20]. The strict classification of MOF and Non-MOF patients accomplished by the scores, which is inevitable for predicting the clinical outcome and statistical analysis, might not be the ideal instrument for daily practice. Preferably, the scores’ use as continuous scale might be helpful with respect to the patients’ daily development.

Recognizing MOF as soon as possible after trauma enables the early assessment of the clinical outcome and the potentially required resource utilisation. Previously, day three after trauma has been shown to be the earliest moment possible defining MOF since organ dysfunction during the immediate posttraumatic treatment may occur due to reversible physiologic derangements [16]. Regarding the overall performance in predicting resource-requiring outcome parameters such as ICU LOS and MVD, the SOFA and Denver Score outperformed the MODS. In patient specific outcome parameter, all scores performed similarly predicting VFD. However, the MODS surpassed the other scores in predicting mortality. The differences in sensitivity and specificity were remarkable, but were also observed in previous studies [14, 17]. For example, due to the Denver score’s low sensitivity, only half of fatal cases were captured in the presented study. However, in the analysed cohort, showing a high rate of severe TBI, not all deaths were associated with or caused by MOF. Still, the differences in sensitivity and specificity of the three scores complicate the understanding which exact clinical picture is meant when speaking of MOF. Harmonising the competing scores and clinical definition would be desirably. Up to that point, comparisons of the scores in different cohorts and with different injury pattern may contribute to the definition.

Certainly, there are difficulties defining this complex syndrome in trauma patients, especially in patients with TBI. The necessity of mechanical ventilation due to thoracic injuries or the required deep sedation due to TBI complicates the scoring of the CNS. Since the GCS is assessed as essential part of the MODS and SOFA score, this grading might lead to false high score values. Furthermore, maintaining a sufficient cerebral perfusion pressure (CPP) often demands the use of vasopressors or inotropic medication, which directly effects the scoring of the cardiovascular system in the SOFA and Denver scores. Using physiologic parameters such as the PAR for grading the cardiovascular system could protect the MODS against therapeutic actions. However, injury pattern and organ dysfunction requires the need of sedation or inotropic medication and therefore contributes to the patients’ overall status displayed by the total score value.

The Denver score was particularly defined and validated in patients without TBI [14, 15]. However, TBI is frequent in European countries with an incidence of 214/100.000 persons/year [21]. In the presented cohort, we observed a TBI incidence of 68 %, which displays the importance of TBI for the daily practice in trauma care. Therefore, we decided deliberately to include these patients and we observed that all three scores work reasonably well in a generalised cohort without excluding this major group of patients.

Considering CNS as a confounder, previous studies have analysed the MODS and SOFA score excluding the GCS scale [12, 22]. Unfortunately, the overall score performance regarding adverse outcomes was not described. Recently Vasilevskis et al. introduced a promising approach substituting the GCS scale by the use of the Richmond Agitation-Sedation Scale (RASS), which is easier to apply in sedated and intubated patients [23, 24]. Although this score has to be validated in a more comprehensive patient cohort including trauma patients, the use of RASS can avoid an underestimation of the SOFA score, which might occur when the neurologic component is ignored [25].

However, some limitations have to be acknowledged. This is a retrospective, single-center study using clinical data. All values have been reassessed for plausibility, but the analysis relies on data documented during the in-patient stay. 84 cases had to be excluded as one or more day three values were missing to apply the scores. In most cases either, patients were not invasive monitored or the GCS was not documented. These missing data might add a selection bias to the presented study. In patients with complete data sets, we used worst daily values for all three scores, although Marshall et al. recommend the first morning values every day to avoid capturing momentary physiological changes [13]. For an improved comparability, we presupposed the same, worst daily values for all three scores as recommended by the SOFA- and Denver score authors [9, 14]. Certainly this might have influenced the MODS’ performance. In the analysed cohort, the cause of death was unfortunately not documented in all cases. Furthermore, not all deaths were associated or caused by MOF. Nevertheless, mortality depicts the poorest possible outcome in trauma patients. Therefore, regardless of the actual cause of death, predicting this fatal clinical course based on daily patient values could be a valuable tool in patient treatment.

## Conclusion

The MODS, Denver and SOFA score have a comparable ability to predict the outcome in severely injured patients including patients with severe TBI. Denver and SOFA score convinced in predicting ICU resource use, while the MODS surpassed the other scores in predicting mortality. The SOFA score showed the most balanced relation between sensitivity and specificity. The incidence of posttraumatic MOF relies decisively on the score applied. Therefore harmonizing the competing scores and definitions would be desirable.

## Abbreviations

AIS: Abbreviated injury scale
ASA: American Society of Anaesthesiologists’ Classification
AUROC: Area under the receiving operating characteristics curve
CI: Confidence intervall
CNS: Central nervous system
CPP: Cerebral perfusion pressure
CVP: Central venous pressure
GCS: Glasgow coma scale
HR: Heart
ICU: Intensive Care Unit
ISS: Injury severity score
LOS: Length of stay
MAP: Mean arterial pressure
MODS: Multiple Organ Dysfunction Score
MODS: Multiple organ dysfunction syndrome
MOF: Multiple organ failure
MVD: Mechanical ventilation days
NISS: New injury severity score
PAR: Pressure-adjusted heart rate
RASS: Richmond Agitation-Sedation Scale
RISCII: Revised injury severity classification II
ROC: Receiver operating characteristics
SBP: Systolic blood pressure
SOFA: Sequential Organ Failure Assessment
TBI: Traumatic brain injury
VFD: Ventilator free days
